# Supplementary figures and images for: A transcriptome-wide association study of uterine fibroids to identify potential genetic markers and toxic chemicals
Source: PLoS One. 2022 Sep 29;17(9):e0274879. doi: 10.1371/journal.pone.0274879 (PMC9521910; doi:10.1371/journal.pone.0274879)

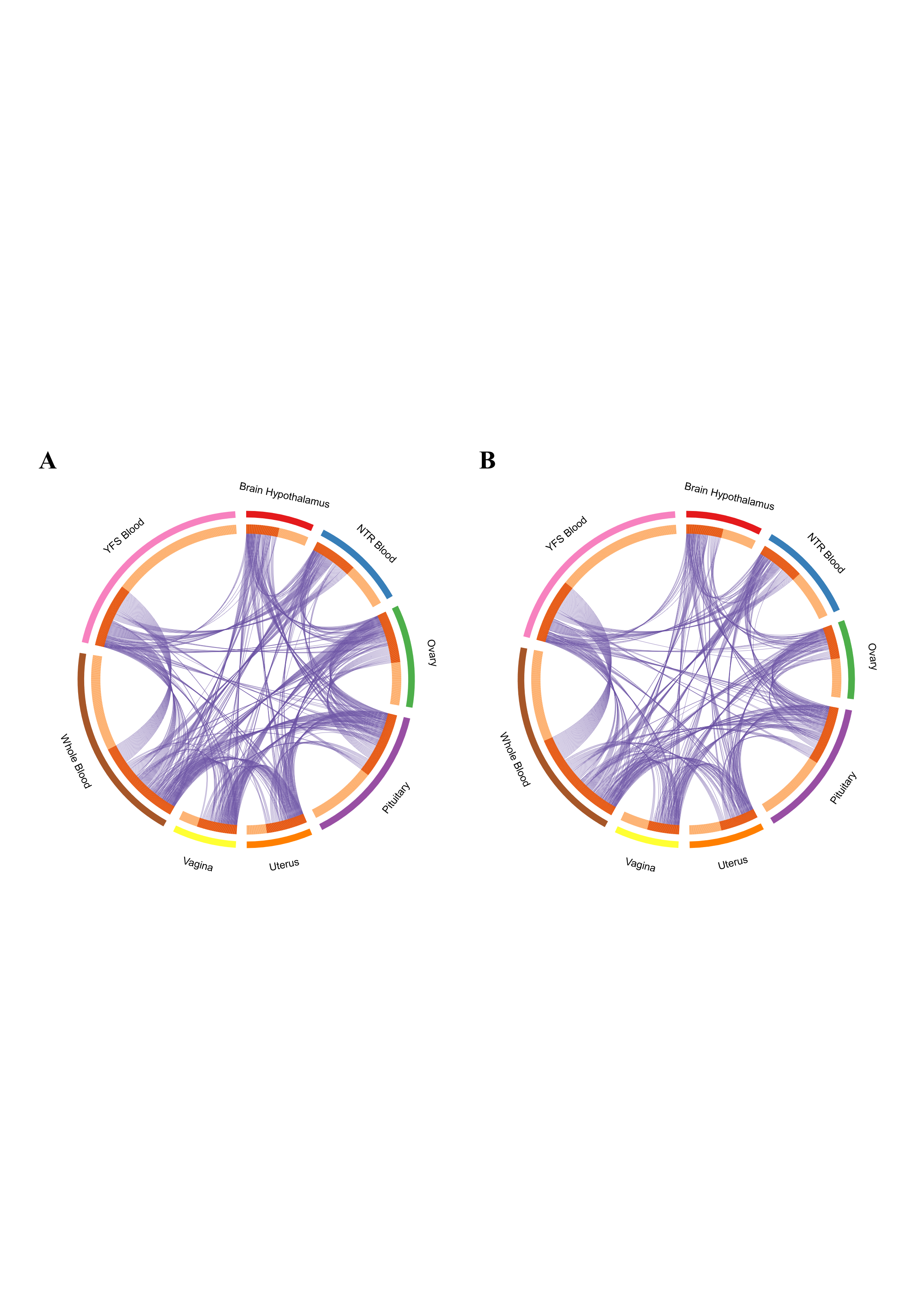

Supplement: S1 Fig — On the outside, the arc represents the eight eQTL panels. On the inside, the dark orange arc represents genes shared by several panels and the light orange arc represents genes unique to those panels. The purple lines link the genes shared by several panels. (A) The plot shows the shared genes between positively associated TWAS genes (TWAS Z-score > 0). (B) The plot shows shared genes between negatively associated TWAS genes (TWAS Z-score < 0). (TIF) [file pone.0274879.s001.tif]

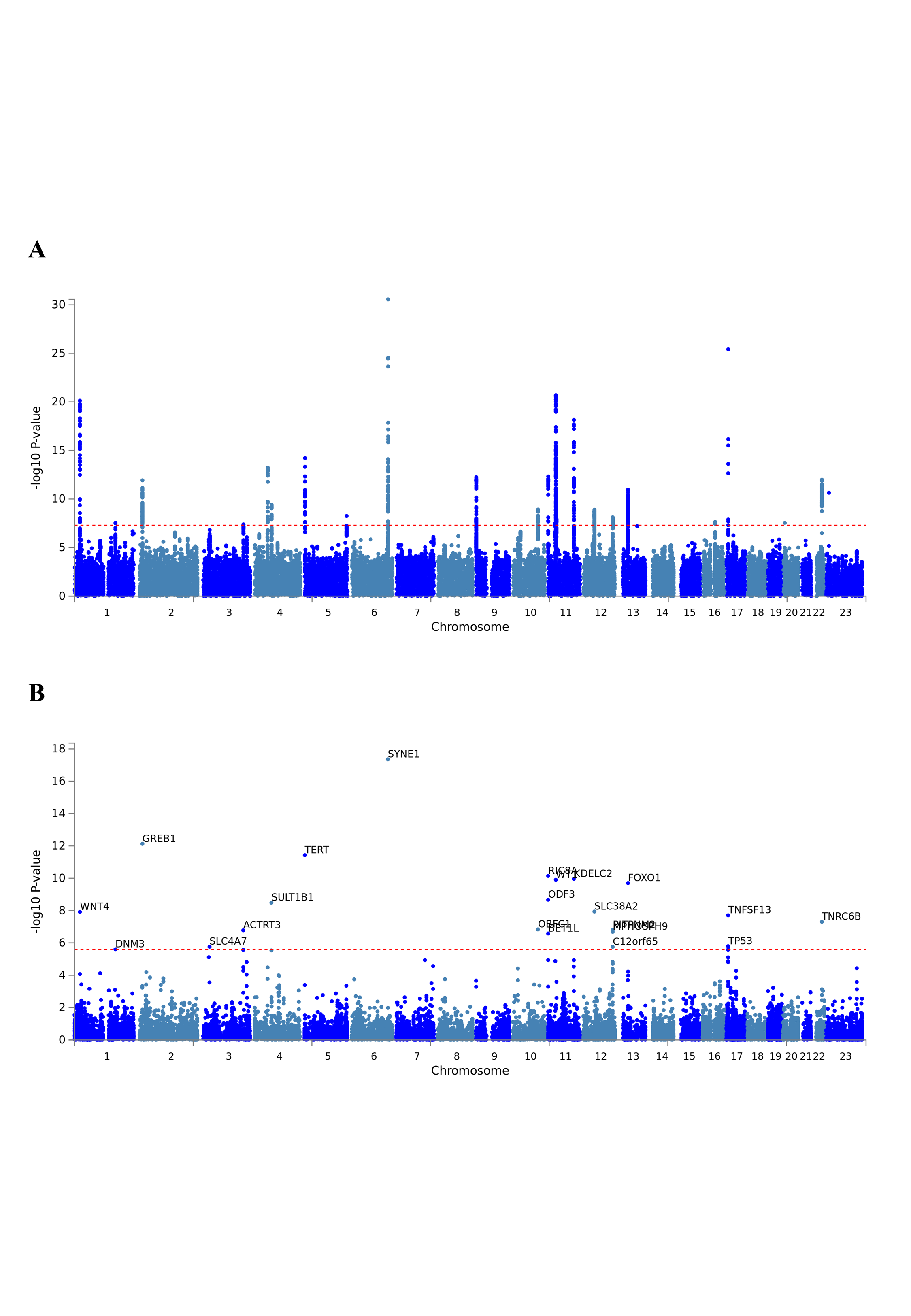

Supplement: S2 Fig — (A) A Manhattan plot of the input GWAS summary statistics. (B) A Manhattan plot of the MAGMA results. Significant prioritized genes associated with SNPs are visualized with gene symbols. The dashed red line indicates a Bonferroni significant threshold (P < 1.90 × 10^−6^). (TIF) [file pone.0274879.s002.tif]

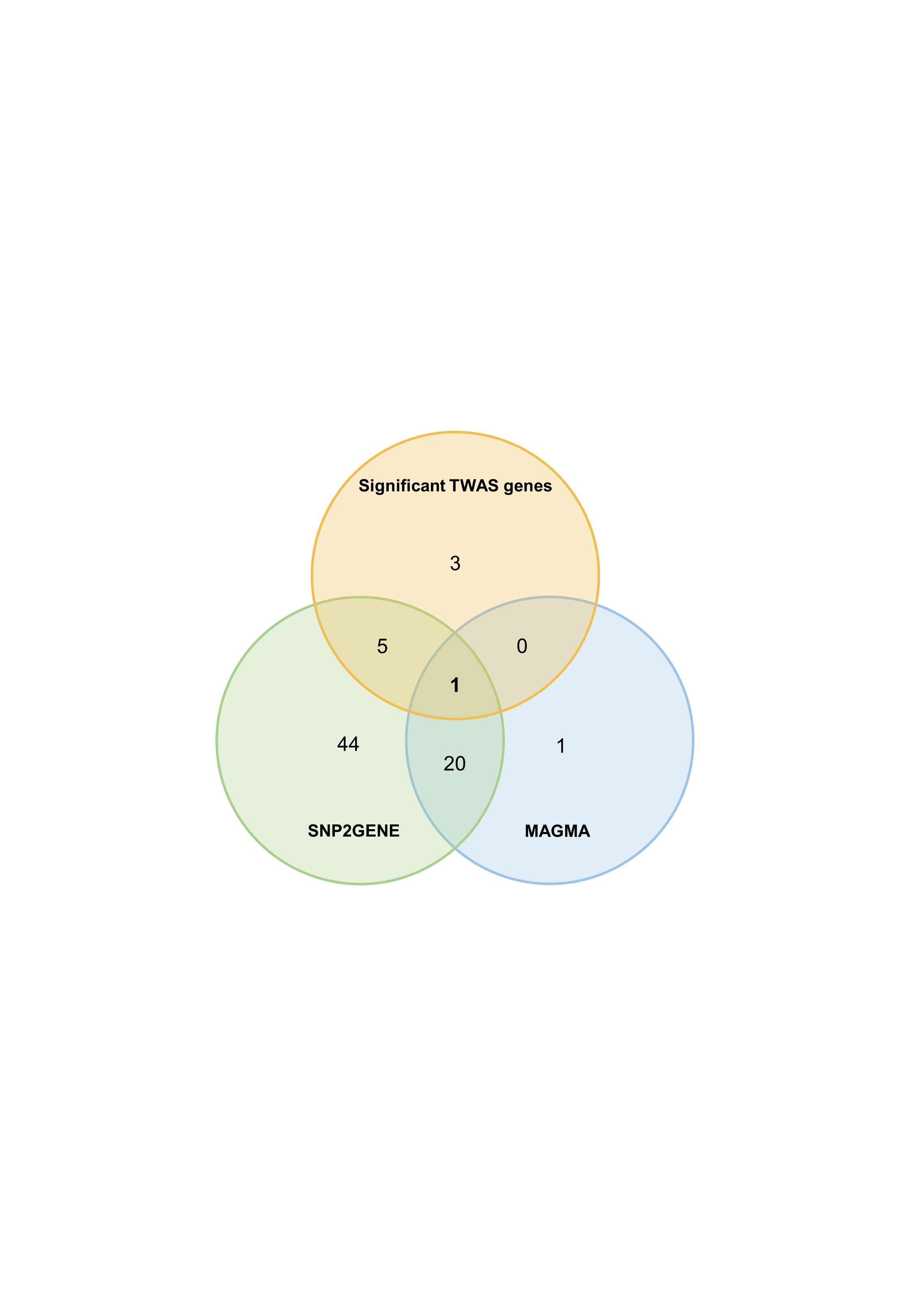

Supplement: S3 Fig — The number of genes only identified in the FUSION is highlighted in bold. (TIF) [file pone.0274879.s003.tif]

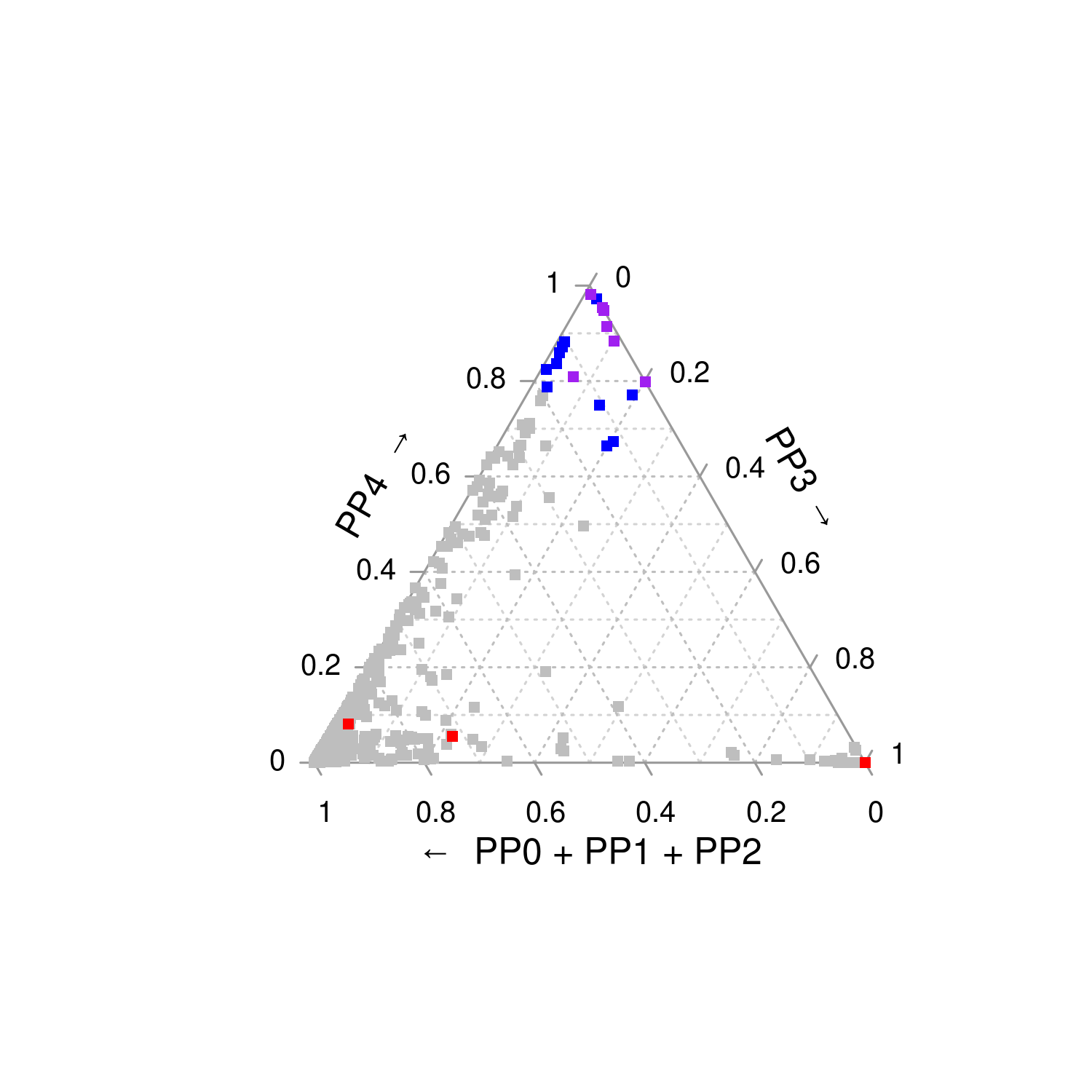

Supplement: S4 Fig — PP0–4 indicate PPs of five hypotheses (H0–4). The gray dots are the genes that were not significant in either TWAS or the colocalization tests. The red and blue dots indicate the significantly associated genes in TWAS and the colocalization tests, respectively. The genes that were prioritized in both TWAS and the colocalization tests are represented as purple dots. (TIFF) [file pone.0274879.s004.tiff]
