# Supplementary material for: A transcriptome-wide association study of uterine fibroids to identify potential genetic markers and toxic chemicals
Source: PLoS One. 2022 Sep 29;17(9):e0274879. doi: 10.1371/journal.pone.0274879 (PMC9521910; doi:10.1371/journal.pone.0274879)
Supplement: Attachment — Submitted filename: Response to Reviewers.docx [file pone.0274879.s013.docx]

**Response to Reviewer #1**

First of all, the authors greatly appreciate the helpful comments that the reviewer made. The authors completely agree with the reviewer’s commentaries and acknowledge that the issues raised by the reviewer should be discussed to improve the quality and objectivity of our study. So the authors tried our best to intensively address the comments to the best of our knowledge. Any modified sentences or phrases are highlighted in the revised version of the manuscript.

Comment 1. Discuss the limitations of the material used: first, the age distribution of the tissue material does not appear suitable, simply because GTEx tissues originate predominately from 50-70 year-old individuals (postmenopausal individuals), while uterine fibroids typically occur premenopausal. Second, discuss limitations of statistical power (see also my next comment)

>>> **Response 1:** The authors appreciate your comment and agree with them. We will add the limitations you pointed out to the discussion along with the issues mentioned in Comment 2.

Comment 2. Discuss the discrepancy between the large number of significant GWAS loci and small number of significant TWAS loci. The former is more than 20 loci (according to Supplementary Fig S4 and many previous UF GWAS studies), while the latter, your novel results, originate from just three distinct loci. - Is it the lack of power in the GTEx material which explains that majority of the GWAS hits do not show TWAS?

>>> **Response 2:** The authors are very grateful for your helpful comment. We fully agree with the need to account for the discrepancy between the number of GWAS loci and TWAS loci. Since the panels used in this study are single-tissue eQTL panels, the statistical power may not be sufficient to capture all true signatures. Also, as mentioned in Comment 1, since the GTEx panels are not composed only for age groups with high UF risk, age-related specificity seems to have decreased. Although it is difficult to immediately solve this problem due to the lack of resources, we believe that more genotype-gene expression associations for UF will be detected in the future if the sample sizes of UF-relevant tissue panels increase or robust multi-content panels are published. We added the limitations to consider as suggested by the reviewers in Comment 1 and 2. Below is the content added to the discussion section (23p, line 468-477).

**The GTEx panels contain a significant proportion of samples from women aged 50**–**70 years who are post-menopausal, whereas the majority of cases of UFs occur in pre-menopausal women. As the tissue panels used in our study did not consist only of age groups with a high UF risk, age-related statistical specificity was reduced. In addition, since the panels used in this study were single-tissue eQTL panels, the statistical power may have been insufficient to detect all true associations. This lack of resources may have contributed to the discrepancy between the number of significant loci in our study and previous UF GWASs. Although it is difficult to address this issue immediately due to a lack of resources, increasing the sample size of UF-related tissue panels or publication of robust multi-content panels may allow the detection of more genotype**–**gene expression associations for UFs.**

**Response to Reviewer #2**

First of all, the authors are grateful for all the helpful comments that the reviewer made. The authors agree entirely with the reviewer’s commentaries and feel that all the comments were necessary. The authors believe that the issues raised by the reviewer were critical to enhance the quality of the study, so the authors tried our best to intensively address the comments to the best of our knowledge. Additionally, while implementing the comments to move some of the content presented in the result section to the discussion section, we have reorganized some descriptions of the result or discussion section for logical structure. Any modified sentences or phrases are highlighted in the revised version of the manuscript.

Comment 1. Lines 48-58: The 2nd paragraph of the introduction mentions genetic risk factors, but then proceeds to predominantly discuss cytogenetic changes observed in fibroid tumors, which are not necessarily risk factors as they can only be observed once a tumor has formed. It may be better to separate the heritability concept from the cytogenetic alterations and instead place it with the germline genetic analyses which are actually evaluating disease risk.

>>> **Response 1:** The authors agree with the reviewer’s comment that it is not appropriate to exemplify the cytogenetic alterations with respect to genetic risk factors for uterine fibroids. To address this issue, we deleted the sentences about studies on cytogenetic alteration and added a reference to reveal the germline genetic risk of uterine fibroid. The second paragraph of the introduction was modified as below (3p, line 48-56).

**Previous studies reported several genetic risk factors affecting UF. A Finnish twin cohort study identified the strong heritability of UF, estimating that monozygotic twins had twice the incidence rate of UF compared with dizygotic twins. Chromosomal abnormalities such as trisomy 12 and rearrangements of chromosomes 12, 13, and/or X are involved in the growth of UF. The overexpression of *High mobility group AT-hook 2*, located in 12q 14–15, is related to the development of UF with or without chromosomal rearrangements. Germline mutations in *fumarate hydratase*, a tumor suppressor gene, stimulate benign and malignant tumor development of UF (1). Deletion of *collagen type Ⅳ alpha 5* and *6 chain* mapped to chromosome X is also known for its association with UF.**

Comment 2. Lines 69-72: The GWAS paragraph includes mention of one GWAS (out of at least 9 that have been performed) and one admixture mapping study, neither of which contain the results which were used in the present manuscript.

>>> **Response 2:** The authors agree that the GWAS paragraph should include the results of the original GWAS study as a reference. We added the results of the Gallagher et al paper to the GWAS paragraph as below (2) (4p, line 70-72).

**A GWAS meta-analysis on UF for European ancestry conducted by Gallagher’s group reported eight risk loci: 2p23.2, 4q22.3, 6p21.31, 7q31.2, 10p11.22, 11p14.1, 12q15, and 12q24.31 (2).**

Comment 3. Line 81: The Gallagher et al paper that produced the GWAS results investigated in this paper but which is not cited directly is not the most recent GWAS.

>>> **Response 3:** In addition to response 2, the authors have confirmed that the GWAS data used in the manuscript is not the most recent and deleted the inappropriate description and changed the sentence as below (4p, line 81-83).

**Herein, we conducted a TWAS by using the meta-analyzed GWAS summary statistics data of UF with eQTL weight panels derived from large-scale consortium data and reference linkage disequilibrium (LD) matrix (2-6).**

Comment 4. Line 115: it is unclear whether the 26,316 represents the total number of unique genes across all 8 tissues, or the sum of genes predicted in each tissue (the technical number of actual tests)

>>> **Response 4:** The authors agree that the number of associations used to set the threshold of statistical significance was unclearly stated. We used the sum of the SNP-gene pair across the tissue panels (26,279) to calculate significance thresholds. We modified the description of the threshold as below (6p, line 117-118).

**(P < 0.05/sum of SNP**–**gene pairs across the tissue panels (26,279) = ~1.90 × 10^-6^).**

Comment 5. Are the joint and conditional tests of TWAS loci performed within FUSION or was there another software/analysis package used for these steps?

>>> **Response 5:** The joint and conditional analyses were performed using the FUSION.post_process.R code provided by FUSION and some visualization code was modified so that readers can visually interpret the results well. We note that there are no critical changes in code which can affect the result of analysis. The method section has been modified to include this description (7p, line 137-139).

**Conditional and joint analyses were conducted to identify independent TWAS genes in a specific locus harboring multiple TWAS associations after conditioning on the expression of TWAS genes using the FUSION.post_process.R code provided by FUSION.**

Comment 6. Line 152: What does CTD stand for?

>>> **Response 6:** It was already explained in line 86 of the introduction section. To simply put, it stands for an abbreviation of comparative toxicogenomics database.

Comment 7. The chemical-gene interaction analysis methods need a bit of rewording to make a couple of items clear. Line 155: chemicals that may induce development of fibroids. Lines 159-160: “the expression caused by the UF” – the gene expressions you have predicted are based on risk OF developing a fibroid, and the tissues are not all from uterus, let alone from the fibroid itself, so this needs to be reworded to be more accurate.

>>> **Response 7:** The authors agree with the reviewer’s comment that we should clarify the chemical-gene interaction analysis method. To solve this issue, we deleted the sentences about obtaining chemicals inducing the development of UF by this method. Line 155 and lines 159-160 of the method were modified as below (8-9p, line 167-172).

**To obtain chemicals that may increase the risk of UF onset, significant TWAS genes with positive or negative Z-scores were respectively provided as input gene sets into the CTD. The chemicals estimated to increase the expression levels of significant TWAS genes with positive Z-scores (TWAS Z-score > 0) or to decrease those with negative Z-scores (TWAS Z-score < 0), in short, chemicals expected to be involved in the pathogenesis of UF, were selected as potentially toxic chemicals for UF.**

Comment 8. The results should indicate that only one of the nine genes was significant in uterus, and that all of the rest were significant in only blood (what is the rationale for including three different whole blood tissue sets? The results do not seem consistent between the three).

>>> **Response 8:** The authors agree that it should be noted that most of the significant associations were discovered in blood panels, so we mentioned this at the result section of the revised manuscript (9-10p, line 189-191).

**Among these nine significant genes, eight genes were detected in one of the three different blood panels (GTEx whole blood, NTR, or YFS), and only *RP11-282O18.3* showed a significant association in the uterus panel.**

>>> Even though all three different blood panels (GTEx, NTR, and YFS) are weight models that predict blood expression, there may be differences between them. By calculating the Pearson’s correlation coefficient between the TWAS.Z values from the three panels, we found up to 27% of discrepancies between the reference expression panels, roughly (Reviewer-only Figure). Based on this, we claim that using all three panels might be helpful to uncover as many potential associations as possible. Several previous TWAS publications, including a study of Gusev’s group, utilized GTEx, NTR, and YFS blood panels for their studies (7-9). In addition, our previous study used the same approach (10). Although it is difficult to find a reference that directly compares GTEx, NTR, and YFS blood panels, GTEx uses RNA-seq expression data, and NTR and YFS use microarray expression data to model the weight panel. In addition, in the aforementioned studies using all of the GTEx, NTR, and YFS blood panels, consistent results were not always obtained in the three blood panels. We added a reference to the method section (6p, line 104-107).

**Reviewer-only Figure. Pearson’s correlation of TWAS Z-score from three blood panels.**

The X and Y axes of each plot indicate the Z-score of the TWAS genes from GTEx, NTR, or YFS blood panels. R represents the coefficient value of Pearson's correlation.

**To uncover as many associations as possible, two different blood panels from individual studies representing whole blood (Young Finns Study, YFS) and peripheral blood (Netherlands Twin Registry, NTR) were also included following previous studies (10).**

Comment 9. It doesn’t appear that any colocalization methods were used to eliminate the potential for LD contamination driving the observed associations in TWAS. This should be considered for robustness.

>>> **Response 9:** The authors greatly appreciate the reviewer for the helpful comment that can enhance the robustness of our study. To address this issue, we additionally performed colocalization tests using the COLOC R package implemented with FUSION (11). Among the 9 TWAS significant genes, 6 were significantly colocalized. The three genes that were not prioritized in the colocalization tests were *LOC653486*, *RP11-282O18.3,* and *KBTBD7*. Although the PP4 (posterior probability that phenotype and gene expression would share the same causal variant) of *LOC653486* was zero, the PP3 (posterior probability that phenotype and gene expression are associated with two different causal variations) of *LOC653486* was 1. This result may delineate the strong relationship between the cis-SNP of *LOC653486* and gene expression. For the other two genes, *RP11-282O18.3* and *KBTBD7*, relatively high PP2s (*RP11-282O18.3* = 0.894, *KBTBD7* = 0.72) were calculated for the association between the individual locus and UF. Additionally, in our original TWAS results, there was no more significant association nearby these two genes. Suppose the TWAS associations for these two genes were inflated by LD contamination. In that case, the PP2 should be lower and the chunk of genes located in the same LD blocks should have shown a significant association in TWAS but this did not happen. Furthermore, conditional and joint analyses performed in our original manuscript showed the independent significance of both genes. Considering these facts, we do not believe that it provided sufficient evidence to reject the potential functional relationship between these two genes and UF.

Even though the additional analysis failed to fully support our original findings using the FUSION software, still, we believe those findings for three genes are meaningful in respect of suggesting the potential association between genotype-mediated gene expression and UF. The results of the colocalization tests were added to the result section and the results were displayed in S3 Table and S4 Fig (12p, line 220-227).

**Colocalization tests were performed to confirm the robustness of the possible causal relationship between the significant TWAS signals and UF. PPs for each TWAS signal associated with UF were calculated by COLOC. The COLOC results showed that six out of the nine significant TWAS genes (*PSMD13*, *MPHOSPH9*, *SBNO1*, *ARL6IP4*, *SETD8,* and *MRPS31*) were replicated in colocalization analyses (PP3+PP4 > 0.8 and PP4/PP3 > 2) (S3 Table and S4 Fig). Together, we identified a total of nine significant TWAS genes including two novel genes—*RP11-282O18.3* and *KBTBD7*—and confirmed the robustness of our results.**

S3 Table. Colocalization test results of COLOC-prioritized and significant TWAS genes.

| **ID** | **PANEL** | **CHR** | **COLOC.PP0** | **COLOC.PP1** | **COLOC.PP2** | **COLOC.PP3** | **COLOC.PP4** | **COLOC_**  **Prioritized** | **IS_TWAS_**  **SIG_GENE** |
| --- | --- | --- | --- | --- | --- | --- | --- | --- | --- |
| *ALDOC* | YFS.BLOOD.RNAARR | 17 | 0 | 0.167 | 0 | 0.009 | 0.824 | Colocalized | NO |
| *ARL14EP* | Ovary | 11 | 0.005 | 0.008 | 0.124 | 0.2 | 0.664 | Colocalized | NO |
| *ARL6IP4* | Whole_Blood | 12 | 0 | 0 | 0.014 | 0.102 | 0.884 | Colocalized | YES |
| *FGFR4* | Whole_Blood | 5 | 0.005 | 0.007 | 0.096 | 0.143 | 0.749 | Colocalized | NO |
| *FOXO1* | YFS.BLOOD.RNAARR | 13 | 0 | 0 | 0.037 | 0.192 | 0.77 | Colocalized | NO |
| *HEATR3* | Ovary | 16 | 0 | 0.142 | 0 | 0.022 | 0.836 | Colocalized | NO |
| *HEATR3* | Uterus | 16 | 0.012 | 0.1 | 0.002 | 0.015 | 0.871 | Colocalized | NO |
| *HEATR3* | Whole_Blood | 16 | 0 | 0.184 | 0 | 0.029 | 0.787 | Colocalized | NO |
| *KBTBD7* | Whole_Blood | 13 | 0.002 | 0.001 | 0.72 | 0.224 | 0.055 | none | YES |
| *LOC653486* | NTR.BLOOD.RNAARR | 11 | 0 | 0 | 0 | 1 | 0 | none | YES |
| *LYG1* | Pituitary | 2 | 0.009 | 0.115 | 0.001 | 0.015 | 0.86 | Colocalized | NO |
| *MPHOSPH9* | Whole_Blood | 12 | 0 | 0 | 0.125 | 0.066 | 0.809 | Colocalized | YES |
| *MPHOSPH9* | YFS.BLOOD.RNAARR | 12 | 0 | 0 | 0 | 0.046 | 0.954 | Colocalized | YES |
| *MRPS31* | YFS.BLOOD.RNAARR | 13 | 0 | 0 | 0 | 0.201 | 0.799 | Colocalized | YES |
| *PSMD13* | NTR.BLOOD.RNAARR | 11 | 0 | 0 | 0.008 | 0.011 | 0.981 | Colocalized | YES |
| *REV1* | Whole_Blood | 2 | 0.001 | 0.103 | 0 | 0.013 | 0.882 | Colocalized | NO |
| *RP11-282O18.3* | Uterus | 12 | 0.003 | 0 | 0.894 | 0.022 | 0.081 | none | YES |
| *SBNO1* | YFS.BLOOD.RNAARR | 12 | 0 | 0 | 0 | 0.052 | 0.948 | Colocalized | YES |
| *SETD8* | Whole_Blood | 12 | 0 | 0 | 0.011 | 0.074 | 0.915 | Colocalized | YES |
| *SLC38A2* | YFS.BLOOD.RNAARR | 12 | 0 | 0 | 0.12 | 0.207 | 0.673 | Colocalized | NO |
| *UIMC1* | YFS.BLOOD.RNAARR | 5 | 0 | 0.001 | 0.001 | 0.026 | 0.972 | Colocalized | NO |

**S4 Fig. A ternary plot of colocalization test results.** PP0–4 indicate PPs of five hypotheses (H0–4). The gray dots are the genes that were not significant in either TWAS or the colocalization tests. The red and blue dots indicate the significantly associated genes in TWAS and the colocalization tests, respectively. The genes that were prioritized in both TWAS and the colocalization tests are represented as purple dots.

>>> The description and details for the colocalization test were described in the method section as below (7p, line 126-134).

**Moreover, we performed colocalization tests with the COLOC R package to determine whether the gene expression signals were colocalized with the GWAS signals (11). COLOC was conducted with FUSION software, and five posterior probabilities (PP0**–**4) were calculated corresponding to five different hypotheses (H0**–**4). H0: no association; H1: functional association but no GWAS association; H2: GWAS association but no functional association; H3: association with gene expression and GWAS signals but each is independent; H4: gene expression and GWAS association are colocalized. Genes satisfying the threshold of PP3+PP4 > 0.8 and PP4/PP3 > 2 were prioritized in the colocalization tests, following previous studies (10, 12).**

>>> Also, in the discussion section, it was mentioned that some genes were not replicated in the colocalization test (24p, line 477-482).

**Even though we identified significant TWAS genes as potential causal genes of UF and confirmed the robustness of this result through the colocalization tests, three genes including our novel findings, *RP11-282O18.3* and *KBTBD7*, were not replicated in the colocalization results. Therefore, the actual effects of our significant TWAS genes on UF risk should be validated through experimental studies.**

Comment 10. The paragraph beginning on line 203 should be moved to discussion, it is not presentation of results.

>>> **Response 10:** The authors agree with the reviewer’s comment that the paragraph beginning on line 203 should be presented in the discussion. The paragraph has been moved to the discussion section of the revised manuscript (18-19p, line 351-373).

Comment 11. How does the conditional analysis handle the differing tissues from which the expression is associated? How were the mentioned SNPs (lines 231, 234, 257) selected? Lead SNPs in the region from FUMA, or known eQTLs for the genes of interest?

>>> **Response 11:** Conditional analyses were carried out for each tissue separately and the results at the same loci were displayed together in a single plot for better visibility. The result section has been modified to include this description (12p, line 233-235).

**The analyses were carried out for each tissue separately and the results at the same locus are displayed together in a single plot for better visibility.**

>> The SNPs mentioned in lines 231, 234, and 257 are lead SNPs of the loci where each significant TWAS gene is located, and they were calculated by FUSION. This information has been added to the legend of Table 1 (11p, line 204-205).

**The LeadGWAS rsID indicates the lead SNPs of the locus where each significant TWAS gene is located, and they were calculated by FUSION.**

Comment 12. Numbers should be included for the numbers of genes included in the up- and down-regulated subsets (lines 273 and 274)

>>> **Response 12:** The authors agree with the reviewer’s comment that the number of genes included in the up- and down-regulated subsets should be clearly presented. Two hundred forty-two genes were included in the up-regulated subsets and 235 genes were included in the down-regulated subsets. This information was added on line 276-278 as below.

**A total of 139 pathways were enriched with the 242 positively associated TWAS genes (TWAS Z-score > 0), while 138 biological pathways were enriched with the 235 negatively associated TWAS genes (TWAS Z-score < 0) (S4 and S5 Tables).**

Comment 13. A large portion of the paragraph beginning on line 299 should be moved to discussion.

>>> **Response 13:** The authors also agree that the paragraph starting from line 299 should be presented in the discussion section. The paragraph has been moved to the discussion section (20-21p, line 388-390, line 396-420).

Comment 14. The statement on line 346 is overstated in the absence of experimental or observational data.

>>> **Response 14:** The authors agree that the expression on line 346 of the manuscript is exaggerated without experimental or observational evidence. To address this, we modified the sentence as below (16p, line 317-319).

**In short, these 66 chemicals may contribute to worsening UF symptoms and/or progression, although the results should be validated by experimental procedures.**

Comment 15. The paragraphs beginning on line 352 and line 360 seem more like discussion.

>>> **Response 15:** The authors agree that the paragraphs beginning on line 352 and line 360 should be stated in the discussion. We modified the manuscript so that those paragraphs are placed in the discussion section of the revised manuscript (21-22p, line 421-449).

Comment 16. The tissues from which the significant results from the previous study’s TWAS came from should be noted.

>>> **Response 16:** The previous TWAS study of Edwards’ group reported *LUZP* from the vagina and *OBFC1* from the esophagus. We added the information to line 333-336 as below.

**A previous study that performed a TWAS using Summary-PrediXcan (S-PrediXcan) software with UF GWAS data from 227,329 samples identified *leucine zipper protein 1* on chromosome 1 from the vagina and *oligonucleotide/oligosaccharide binding fold containing 1* on chromosome 10 from the esophagus as potential causal genes for UF (13).**

Comment 17. A 7% sample size increase at the scale of 200 thousand participants seems like a stretch to claim increased power for detection. It is notable that the 5 of the 10 significant results came from tissue sources not analyzed in the previous publication, and likely that the differing software and reduced multiple-testing burden in your paper also contributed to the differences in findings.

>>> **Response 17:** The authors agree with the reviewer’s comment that the interpretation of the difference between the previous study and ours was somewhat inflated. We revised the discussion section about the possible causes of discrepancies from previous publications as commented by the reviewer. The paragraph beginning with line 337 includes that content.

**Even though S-PrediXcan reportedly shows results consistent with those of FUSION, our study differs from the previous TWAS in a few ways. The previous study utilized S-PrediXcan as the TI method with every tissue-specific panel of GTEx v7 excluding male-specific tissues. We performed TI using FUSION with UF-related tissue panels including two blood panels from NTR and YFS that were not analyzed in the previous publication, because our main focus was potentially strong tissue-specific regulatory effects on the pathogenesis of UF. The reduction in the multiple-testing burden resulting from the use of fewer tissue panels may have contributed to identifying novel putative causal genes for UF that were previously undetected**.

Comment 18. The sentence on lines 426-427 doesn’t make sense.

>>> **Response 18:** The authors agree that the sentence on lines 426-427 presented to summarize the results of functional annotation may be difficult for readers to understand. To solve this problem, it has been modified as below (20-21p, line 404-406).

**Some of the other TWAS genes positively associated with UF were found to be enriched in pathways belonging to the parental pathway ‘localization’**.

Comment 19. The paragraph beginning on line 436 has 10 lines about a chemical you excluded from results, this seems unnecessary to interpretation of the current results.

>>> **Response 19:** The authors totally agree that we do not have to interpret a chemical we excluded from the results. We deleted lines 436-446 and modified line 421 as below.

**Using the CTD, we identified five toxic chemicals associated with our TWAS genes expected to be involved in the pathogenesis of UF (Table 2).**

Comment 20. The sentence on lines 464-465 recommending that women with certain genetic profiles should avoid dietary chemical exposures is a complete overstatement. At MOST this should be presented as a relationship which should be evaluated in model organism experimental or human observational studies well before guidelines should be presented to patients, who most likely do not know what the risk “genetic backgrounds of UF” are, let alone whether they carry such profiles.

>>> **Response 20:** The authors are truly grateful for the reviewer’s comment. We totally agree that the sentence recommending that women with certain genetic backgrounds of UF should avoid dietary chemical exposures was overstated. We modified the sentence by suggesting the possibility that chronic dietary exposure to aflatoxin B1 and BPDE aggravate symptoms and/or progressions of UF and the necessity of experimental study that the exposure to chemicals affects the pathogenesis of UF as below (23p, line 454-458).

**Based on our results, women with certain genetic backgrounds of UF due to chronic dietary exposure to aflatoxin B1 and/or BPDE may show a higher risk of UF; although the actual effects of the exposure should be validated through experimental studies, it is also true that exposure to aflatoxin B1 and BPDE should always be avoided because they are toxic.**

**Reference**

1.	Tomlinson IP, Alam NA, Rowan AJ, Barclay E, Jaeger EE, Kelsell D, et al. Germline mutations in FH predispose to dominantly inherited uterine fibroids, skin leiomyomata and papillary renal cell cancer. Nat Genet. 2002;30(4):406-10.

2.	Gallagher CS, Makinen N, Harris HR, Rahmioglu N, Uimari O, Cook JP, et al. Genome-wide association and epidemiological analyses reveal common genetic origins between uterine leiomyomata and endometriosis. Nat Commun. 2019;10(1):4857.

3.	Consortium GT, Laboratory DA, Coordinating Center -Analysis Working G, Statistical Methods groups-Analysis Working G, Enhancing Gg, Fund NIHC, et al. Genetic effects on gene expression across human tissues. Nature. 2017;550(7675):204-13.

4.	Wright FA, Sullivan PF, Brooks AI, Zou F, Sun W, Xia K, et al. Heritability and genomics of gene expression in peripheral blood. Nat Genet. 2014;46(5):430-7.

5.	Raitakari OT, Juonala M, Ronnemaa T, Keltikangas-Jarvinen L, Rasanen L, Pietikainen M, et al. Cohort profile: the cardiovascular risk in Young Finns Study. Int J Epidemiol. 2008;37(6):1220-6.

6.	Genomes Project C, Auton A, Brooks LD, Durbin RM, Garrison EP, Kang HM, et al. A global reference for human genetic variation. Nature. 2015;526(7571):68-74.

7.	Gusev A, Mancuso N, Won H, Kousi M, Finucane HK, Reshef Y, et al. Transcriptome-wide association study of schizophrenia and chromatin activity yields mechanistic disease insights. Nat Genet. 2018;50(4):538-48.

8.	Lindstrom S, Wang L, Smith EN, Gordon W, van Hylckama Vlieg A, de Andrade M, et al. Genomic and transcriptomic association studies identify 16 novel susceptibility loci for venous thromboembolism. Blood. 2019;134(19):1645-57.

9.	Dall'Aglio L, Lewis CM, Pain O. Delineating the Genetic Component of Gene Expression in Major Depression. Biol Psychiatry. 2021;89(6):627-36.

10.	Song J, Kim D, Lee S, Jung J, Joo JWJ, Jang W. Integrative transcriptome-wide analysis of atopic dermatitis for drug repositioning. Commun Biol. 2022;5(1):615.

11.	Giambartolomei C, Vukcevic D, Schadt EE, Franke L, Hingorani AD, Wallace C, et al. Bayesian test for colocalisation between pairs of genetic association studies using summary statistics. PLoS Genet. 2014;10(5):e1004383.

12.	Li YI, Wong G, Humphrey J, Raj T. Prioritizing Parkinson's disease genes using population-scale transcriptomic data. Nat Commun. 2019;10(1):994.

13.	Edwards TL, Giri A, Hellwege JN, Hartmann KE, Stewart EA, Jeff JM, et al. A Trans-Ethnic Genome-Wide Association Study of Uterine Fibroids. Front Genet. 2019;10:511.
